# Extraperitoneal Single-Port Robot-Assisted Radical Prostatectomy for a Massive Prostate: A Case Report

**DOI:** 10.7759/cureus.87771

**Published:** 2025-07-12

**Authors:** Hiroki Ishikawa, Masaru Ishida, Tadatsugu Anno, Tansei Sanjo, Masashi Arai

**Affiliations:** 1 Urology, Saiseikai Yokohamashi Tobu Hospital, Yokohama, JPN

**Keywords:** da vinci surgical system, prostate cancer, robotic-assisted surgery, robotic prostatectomy, single-port robotic-assisted surgeries

## Abstract

Extraperitoneal single-port robot-assisted radical prostatectomy (SP-RARP) using the da Vinci SP system (Intuitive Surgical, Sunnyvale, CA, US) offers a less invasive alternative to multi-port RARP. However, a significant limitation is the system’s reduced instrument force, which makes retraction and manipulation of large, heavy organs particularly challenging. As a result, SP-RARP for prostates over 100 g has been considered difficult and is rarely reported. This case highlights the feasibility of extraperitoneal SP-RARP in a patient with a massive prostate by addressing and overcoming this technical limitation.

We report the case of a 77-year-old man with bilateral ureteral stones, significant prostatic hypertrophy, and prostate adenocarcinoma (Gleason score 3+3), who underwent extraperitoneal SP-RARP due to urinary retention. The operation lasted 4 hours and 40 minutes, with an estimated blood loss of 450 mL through a 4.5 cm sub-umbilical incision. The prostate weighed 143 g. No intraoperative complications occurred, and the postoperative course was uneventful.

SP-RARP for massive prostates (>100 g) remains technically challenging due to the limited retraction force of the single-port system, and successful extraperitoneal cases have not been previously documented. This report demonstrates that, with proper technique, extraperitoneal SP-RARP can be safely and effectively performed even in cases involving significantly enlarged prostates. These findings support the potential expansion of SP-RARP indications in select high-volume cases.

## Introduction

Single-port robot-assisted radical prostatectomy (SP-RARP) is increasingly recognized as a less invasive alternative to multi-port RARP. However, few case reports or large-scale studies have examined this technique. SP-RARP can be performed via either a transperitoneal or extraperitoneal approach, with the latter often preferred due to its association with fewer complications and shorter hospital stays. A known limitation of the da Vinci SP system (Intuitive Surgical, Sunnyvale, CA, USA) is the reduced strength of its instruments compared to multi-port systems. This weaker retraction force poses a significant challenge when manipulating large, bulky tissues, a critical step in prostatectomy. As a result, SP-RARP for prostates larger than 100 g is rarely attempted, as the limited surgical field and inadequate instrument force make such procedures technically demanding [1]. This case report describes a successful extraperitoneal SP-RARP for a prostate weighing over 100 g, demonstrating the approach’s feasibility and contributing important evidence to the growing body of literature.

## Case presentation

**Figure 1:**
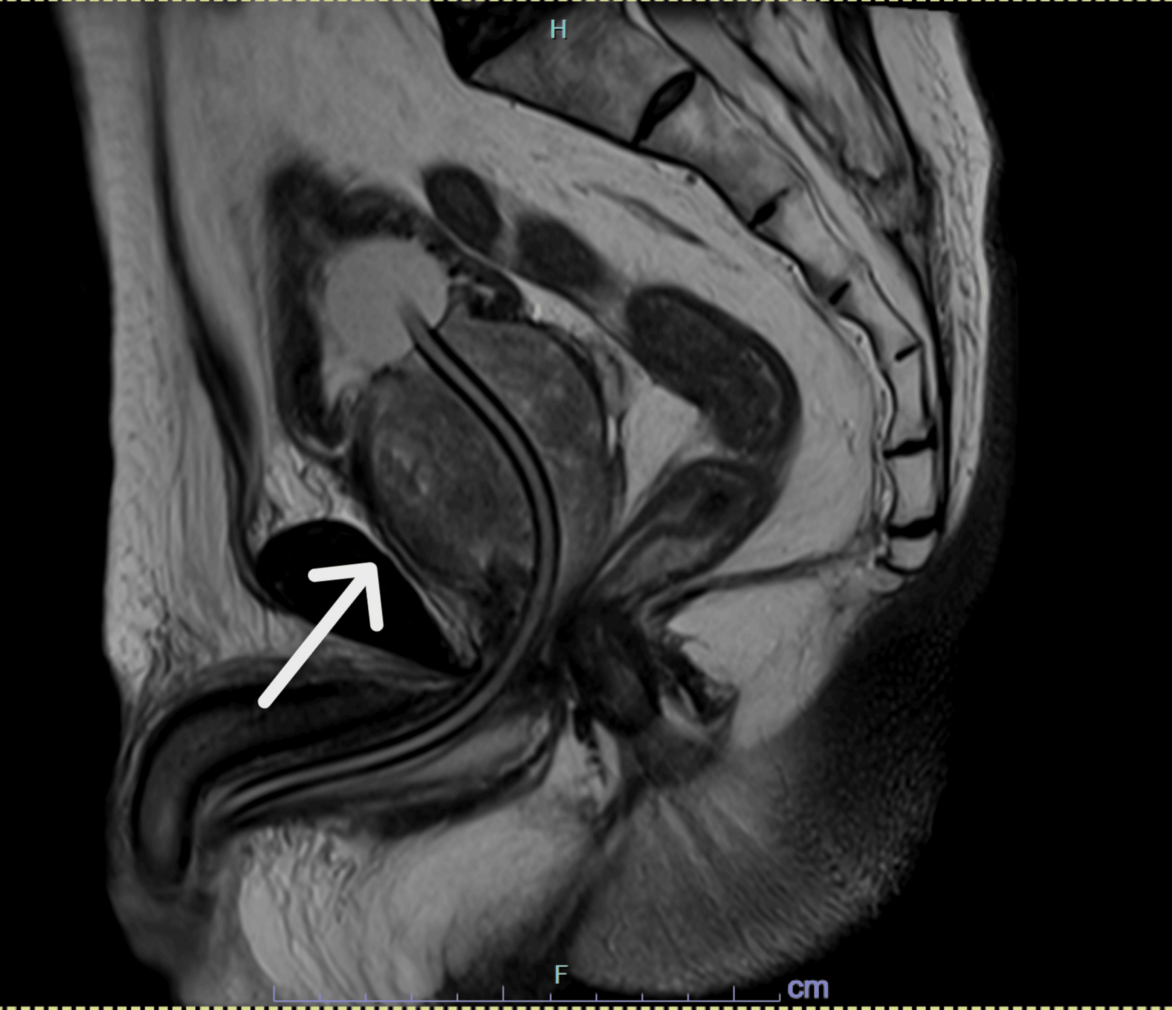
MRI T2 sagittal view of the massive prostate when a Foley catheter was placed

**Figure 2:**
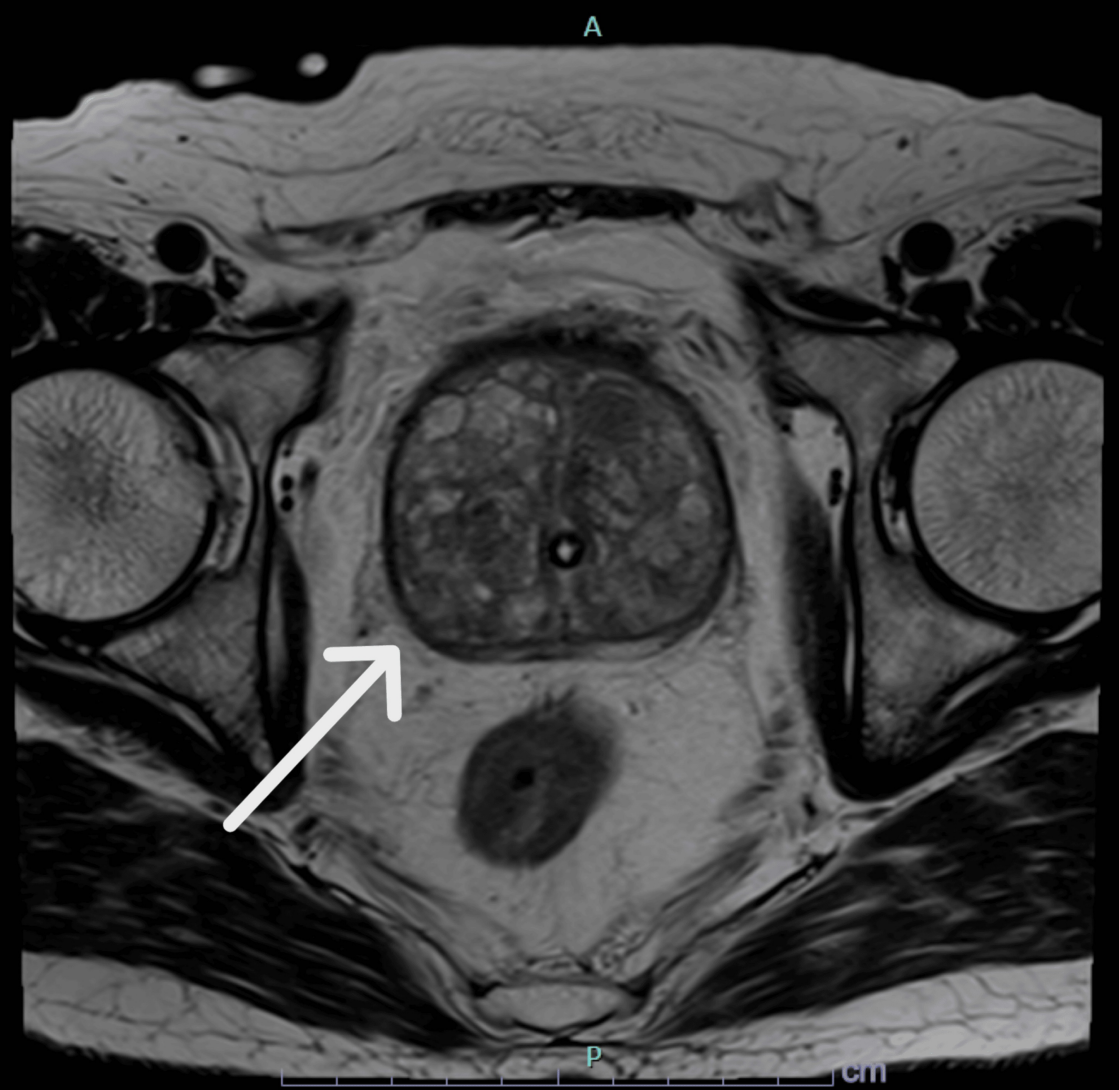
MRI T2 axial view of the massive prostate when a Foley catheter was placed

A 77-year-old male (height: 163 cm, weight: 68.7 kg, body mass index (BMI): 25.8 kg/m^2^) with a medical history of hypertension, hyperlipidemia, and urolithiasis presented with bilateral flank pain. Computed tomography (CT) confirmed bilateral ureteral stones (right: 7 × 7 mm, left: 9 × 8 mm) along with significant prostatic hyperplasia. His serum creatinine was elevated at 2.35 mg/dL (normal range: 0.65-1.07 mg/dL), indicating postrenal renal failure, for which bilateral ureteral stents were placed. His prostate-specific antigen (PSA) level was markedly elevated at 24.80 ng/mL (normal range: 0.0-2.7 ng/mL). Multiparametric magnetic resonance imaging (MP-MRI) estimated the prostate volume at 115 mL and showed no clear signs of malignancy (Figures 1, 2). However, transrectal ultrasound-guided biopsy revealed adenocarcinoma (Gleason score 3+3) in 4 out of 10 cores. Given his recurrent urinary retention and overall good health (ASA Class II), RARP was planned.

**Figure 3:**
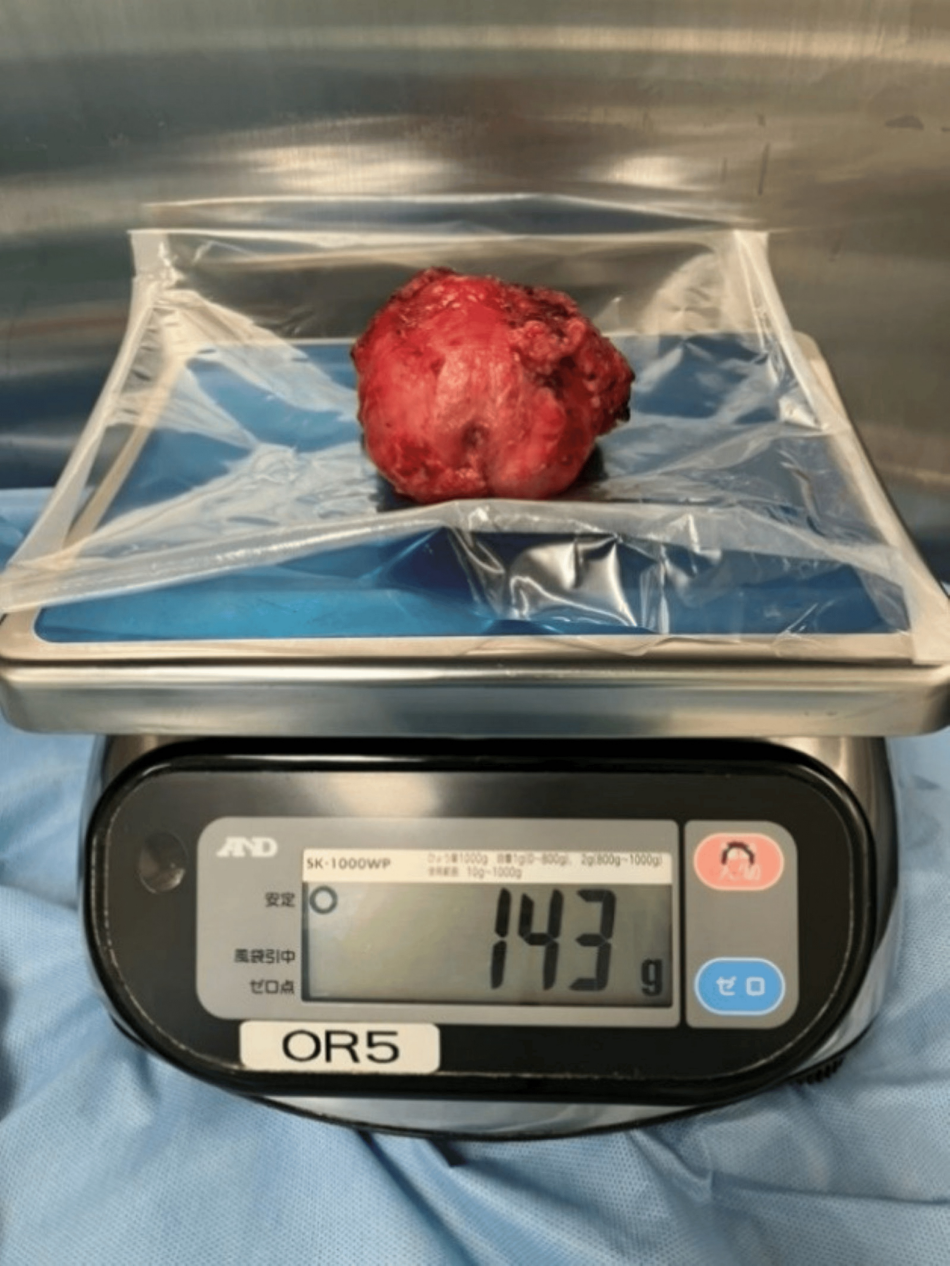
The resected prostate, weighing 143 g

Extraperitoneal SP-RARP was performed through a 4.5 cm transverse sub-umbilical incision. An additional 12 mm assistant port was placed in the right lower abdomen. The patient was positioned in a 10° Trendelenburg position. Surgical instruments included a fenestrated bipolar grasper, Cadiere forceps, and monopolar curved scissors, with the camera positioned at the 12 o'clock orientation. Due to the large prostate, the neurovascular bundles (NVBs) were significantly elongated, requiring meticulous dissection and precise hemostasis. The console time was 3 hours and 59 minutes, with a total operative time of 4 hours and 40 minutes. Estimated blood loss was 450 mL, and the resected prostate weighed 143 g (Figure 3).

The drain was removed on postoperative day (POD) 2. A cystogram performed on POD 4 confirmed no urinary leakage, and the Foley catheter was subsequently removed. The patient was discharged on POD 5. Preservation of sexual function was not attempted due to NVB dissection.

At two months postoperatively, the patient required three pads per day, indicating relatively good urinary continence. His prostate-specific antigen (PSA) level was <0.01 ng/mL, suggesting a favorable early biochemical response.

## Discussion

SP-RARP has been performed using various surgical approaches. Among these, the extraperitoneal SP-RARP has shown benefits in reducing postoperative pain, complications, and hospital stays compared to conventional multi-port and transperitoneal RARP [2,3]. It is expected to become increasingly adopted as a less invasive and advantageous option for patients. However, large-scale studies on extraperitoneal SP-RARP remain limited. Review of previous reports reveals no documented cases of extraperitoneal SP-RARP involving resection of prostates exceeding 100 g, with reported gland sizes ranging only from 15 g to 62 g [1,4,5].

The da Vinci SP system has several limitations compared to the Xi system. Most notably, it provides reduced instrument strength, resulting in significantly weaker retraction capability. During prostatectomy, adequate elevation of the prostate is essential for posterior dissection and NVB management. The SP system’s limited instrument force makes manipulation of large prostates particularly challenging, which has contributed to its primary use in patients with smaller prostate volumes [1]. Additionally, the confined extraperitoneal workspace compounds motion constraints and contributes to a steeper learning curve for surgeons [6,7].

The surgeon in this case had prior experience with 343 RARP procedures, including 56 with the da Vinci S system, 282 with the Xi system, and 5 with the SP system. Despite the technical challenges inherent in this approach, the surgeon successfully completed extraperitoneal SP-RARP on a prostate weighing 143 g, demonstrating the feasibility of this technique even in cases involving massive prostate enlargement.

Key surgical adaptations are detailed below. Due to limited exposure of the posterior prostate, dissection of the NVB and posterior prostate was performed alternately in an antegrade fashion. The extended distal articulation of the SP instruments (longer than those of the Xi system) was used to lift the prostate. Given the specimen’s size, docking was temporarily released to allow external extraction. After partial closure of the rectus fascia, the port was reinserted, and surgery resumed. Although suturing and suspending a significantly enlarged middle lobe to the abdominal wall can assist in similar cases, this was unnecessary here. Preoperative bilateral ureteral stents, placed for the patient’s ureteral stones, helped minimize the risk of ureteral injury during bladder neck reconstruction and vesicourethral anastomosis. The limited retraction force and narrow pelvic space necessitated careful cold dissection during NVB separation to avoid rectal injury. Clip usage was minimized to prevent inadvertent rectal clipping.

To our knowledge, this is the first reported case of extraperitoneal SP-RARP for a prostate of this size, directly addressing concerns about the SP system’s limited instrument strength. Although the procedure duration was extended, this case demonstrates that thoughtful technical adjustments can overcome the system’s inherent limitations and expand the indications of SP-RARP. Notably, postoperative analgesia was limited to five days of acetaminophen. Under Japan’s health insurance system, patient cost remains the same regardless of a 1-2 day versus 5-day hospital stay. Thus, despite the longer admission period (until catheter removal), the case qualifies as a minimally invasive procedure in terms of pain control. Further research is warranted to evaluate the long-term oncological and functional outcomes of SP-RARP, particularly regarding sexual function and urinary continence, to better define its role in treating patients with massive prostates.

## Conclusions

While SP-RARP is often considered unsuitable for massive prostates, we successfully performed the procedure on a prostate exceeding 100 g without significant complications. This case demonstrates that the challenge of limited retraction associated with the SP system can be effectively managed through appropriate surgical strategies and experience. The indications for SP-RARP should not be overly limited by the system’s technical constraints, as its application can be expanded with well-planned techniques, even in cases involving large prostate glands.
